# Wastewater-Irrigated Vegetables Are a Significant Source of Heavy Metal Contaminants: Toxicity and Health Risks

**DOI:** 10.3390/molecules28031371

**Published:** 2023-02-01

**Authors:** Kiran Aftab, Sarosh Iqbal, Mohammad Rizwan Khan, Rosa Busquets, Razia Noreen, Naushad Ahmad, Syed Gohar Taqi Kazimi, Abdulnasser Mahmoud Karami, Nouf Mohammad Saad Al Suliman, Mohamed Ouladsmane

**Affiliations:** 1 Department of Chemistry, Government College University, Faisalabad 38000, Pakistan; 2 Department of Applied Chemistry, Government College University, Faisalabad 38000, Pakistan; 3 Department of Chemistry, College of Science, King Saud University, Riyadh 11451, Saudi Arabia; 4 School of Life Sciences, Pharmacy and Chemistry, Kingston University London, Kingston Upon Thames KT1 2EE, UK; 5 Department of Biochemistry, Government College University, Faisalabad 38000, Pakistan; 6 Department of Chemistry, University of Sargodha, Sargodha 40100, Pakistan

**Keywords:** heavy metals, bioconcentration factor, metals intake, health risk, wastewater-irrigation

## Abstract

Water contaminated with heavy metals constitutes an important threat. This threat is a real problem with a negative impact in some developing countries where untreated industrial effluents are used for irrigation. The present study examines heavy metals in wastewater-irrigated vegetables (apple gourd, spinach, cauliflower, sponge gourd, and coriander) water, and soil from Chenab Nagar, Chiniot, Pakistan. In particular, the metals quantified were cadmium (Cd), chromium (Cr), cobalt (Co), nickel (Ni), lead (Pb), and manganese (Mn). Among them, Cr and Co in crops irrigated -wastewater exceeded the levels recommended by the World Health Organization (WHO). In contrast, Ni, Cu, Pb, and Mn concentrations were in line with WHO standards. Compared with the limits established by the Food and Agriculture Organization of the United Nations (FAO), all the study vegetables presented higher (thus unsafe) concentrations of Cd (0.38 to 1.205 mg/Kg). There were also unsafe concentrations of Cr in coriander, sponge gourd, and cauliflower. Pb was found at an unsafe concentration (0.59 mg/Kg) in cauliflower. Conversely, Ni and Mn concentrations were below the maximum permissible limits by WHO, and FAO in all of the analyzed samples. The contamination load index (CLI) in soil, bioconcentration factor (BCF) in plants, daily intake of metals (DIM), and health risk index (HRI) have also been evaluated to estimate the potential risk to human health in that area. We have found an important risk of transitions of Pb, Cd, Cr, and Co from water/soil to the edible part of the plant. The highest HRI value associated with Cd (6.10–13.85) followed by Cr (1.25–7.67) for all vegetable samples presented them as high health risk metal contaminants. If the issue is not addressed, consumption of wastewater-irrigated vegetables will continue posing a health risk.

## 1. Introduction

Organisms and soils regulate their concentrations of metals and allied compounds. In recent years, however, anthropogenic activities have increased metal release into the environment, and some of these metals disrupt biogeochemical cycles [1,2]. Consequently, heavy metal contamination in water sources is becoming a major concern in the developing world [3,4]. Major sources of heavy water contamination are industrial growth, volcanic eruptions, forest fires, corrosion, mining, sewage sludge, and agrochemicals [4]. The fate of metals is diverse [5]. Metal sorbed in soil and contaminating water can be transferred to plants, which are then consumed by livestock or humans and can harm their well-being [6,7]. Trace amounts of various metals, such as cobalt (Co), manganese (Mn), lead (Pb), and chromium (Cr) are very important in biological processes. Nevertheless, when essential metals exceed certain levels, these become toxic to organisms [8,9,10]. Metals such as cadmium (Cd), mercury (Hg), tin (Sn), and arsenic (As) have no biological role and are hazardous for the environment even when available in small quantities or above the maximum recommended limits [8,11,12,13]. Many developing countries, including India, Iran, Egypt, Bangladesh, and Pakistan are experiencing heavy metal contamination in their flora and fauna as a result of rapid urban and industrial developments. This directly impacts food quality and the environment and can impact the health of their citizens [14,15,16,17]. 

The consumption of vegetables is an important component of a balanced diet [18,19]. Besides offering an inexpensive source of nutrition, vegetables provide minerals, folic acid, niacin, pyridoxine, thiamine, vitamins, and dietary fiber. Fruits and vegetables play a role in preventing oxidative damage through the inhibition of free radicals [20,21,22,23,24,25,26]. However, crops grown in contaminated water can reach toxic levels of metals. Hencelong-term consumption of polluted vegetables may result in severe health problems affecting bones, the cardiovascular and nervous systems, kidneys, and liver [5,27,28,29,30]. Hence, monitoring heavy metal concentrations in crops is necessary to know the extent of the threat. Methods that include the analysis of bio-indicators are used to measure the effects of harmful substances on living organisms [31,32]. In this study, heavy metals such as Cd, Cr, Co, Pb, nickel (Ni), and manganese (Mn) were assessed in wastewater-irrigated vegetables located on the east bank of the Chenab river (31.307 N, 72.328 E), Chiniot, Pakistan. The city has an extreme climate with hot temperatures with significant variations during summer and winter. The transfer of these heavy metals to the food chain was investigated. Additionally, indices calculations for instance CLI, BCF, DIM, and HRI were also made in order to estimate the potential risk to livestock consuming those vegetables (foodstuff) in that area.

## 2. Results and Discussion

### 2.1. Heavy Metals in Wastewater Used for Irrigation and Soil

The concentration of heavy metals in wastewater used for irrigating crops and soil samples from the irrigated study area are given in Table 1. Pb was not detected in irrigated-wastewater, although it was detected in in soil (0.11 mg/Kg). Its origin in soil could be from past contamination from water or atmospheric deposition via dust. 

According to WHO (2014), Cd, Cr, Co, Ni, Mn, and Pb should not exceed 0.01, 0.1, 0.1, 0.20, 0.20, and 5.0 mg/L in wastewater used for irrigation [33]. Nevertheless, the observed concentrations of Cr and Co in wastewater used for irrigation exceeded these safe limits and further work is needed to track their origin. This could result from the furniture industry in Chiniot, as Cr is a constituent of wood-preservative formulations, while Co and Pb are main constituents of coating pigments [34]. The levels of Cd, Ni, Mn and Pb in wastewater for irrigation were within the WHO’s recommended limits [32]. 

In accordance with USEPA [29], the maximum permissible limits for Cd, Cr, Ni, and Pb in soil are 3, 100, 50, and 2000 mg/kg, respectively. As shown in Table 1, the levels found for all these metals were found to be safe (below maximum permissible limits). 

### 2.2. Heavy Metals in Vegetables 

A summary of the concentrations of heavy metals in edible parts of vegetables is presented in Table 2. The mean concentration of Cd spans from 0.38 to 1.20 mg/kg. According to FAO/WHO standards for vegetables, for bulbs and fruits, up to 0.05 mg Cd/kg is acceptable, while for leafy and tuberous vegetables, 0.1 mg/kg is the limit [35]. Therefore, the mean concentration of Cd in all samples in this study exceeded safe limits. Understanding the fate of Cd in vegetables is important for food safety. Previous studies found that Cd accumulates primarily in leaves, whereas seeds, fruit, and roots usually contain lower levels [36]. The greatest contamination of Cd was found in apple gourd and spinach leaves, followed by cauliflower, sponge gourd, and coriander among eatable parts of plants studied in our work [37,38,39]. In earlier studies, the researchers have also reported higher Cd concentrations in soil, crops, and intensive greenhouse vegetable production systems along the Yellow Sea of China [40,41]. Our results are in agreement with works where fruit and leaves had greater levels of Cd than stems. Overall, Cd accumulates more in the above-ground parts and does not become immobilized in roots [42]. 

The presence of Cr found in vegetables covered a broader range of concentrations (0.72–4.00 mg/kg) than Cd. The amount of Cr in spinach fruit (3.45 mg/kg); cauliflower leaves (2.49 mg/kg), stem (2.26 mg/kg) and fruit (2.91 mg/kg); sponge gourd leaves (2.52 mg/kg), stem (2.52 mg/kg) and fruit (4.00 mg/kg); and coriander leaves (4.00 mg/kg) and stem (3.80 mg/kg) were at higher levels than the safe limit recommended by the FAO/WHO standards for vegetables [35]. The concentration of Cr in edible parts of coriander, sponge gourd, and cauliflower was all above the safe limit (2.3 mg/kg), hence Cr is a problematic metal that requires further investigation and remediation work in the study area.

Co was between 0.76 mg/kg and 2.08 mg/kg in vegetables. The uptake was maximum of 2.08 mg/kg in cauliflower fruit, however, all the vegetables had Co concentration below the FAO/WHO recommended level of 50 mg/kg of vegetables [35]. The concentration of Co in vegetables in the present study is in line with the result reported in a previous study [43] and would not cause toxic effects [44].

According to the FAO/WHO, Ni, Mn and Pb accumulation in plants should not exceed 67.9, 500, and 0.3 mg/kg [35]. The values of trace metals (Ni, Mn) in all samples were found lower than the permissible limits except for Pb (at 0.59 mg/kg) was observed greater than in the edible part of cauliflower. The permissible Pb concentration is up to 0.1 mg/kg in fruit, tuberous and bulbous vegetables, and 0.3 mg/Kg in leafy vegetables. A possible reason for high Pb concentration in vegetables and soil could be deposition from emissions from traffic. Indeed, Pb was not detected in wastewater (see Table 1), and concentrations were within safe limits in soil, although accumulation from water or soil could take place. Variations in the availability of metals to plants may be attributed to differences in the absorption, competition, and uptake mechanisms of metal ions. Interaction among heavy metals may also take place and these mainly occur near the root surface [45]. The accumulation of metals in plants will also be affected by plant age. In leafy and tuberous plants, metal concentrations were greater compared to fruit vegetables. This pattern was in line with previous studies [30].

The statistical significance of variations in vegetable samples (Table S1) resulted from a two-way Analysis of Variance (ANOVA) determined the statistical significance (α= 0.05) of metals ions concentrations in different vegetables. The calculated *p*-value (Tukey test) among different parts (stem, leaves, fruit/flower) of apple gourd, spinach, cauliflower, sponge gourd, and coriander are less than zero which indicates the highly significant variation in all studied metals among all parts of vegetables under investigation. Table S1 in supporting information provides the specific statistical values evidencing the significant differences observed among metals in parts of plants. The effect of heavy metals in the variation of results in the different vegetable parts was highly significant for apple gourd (*p* 0.0046); cauliflower (*p* 0.0072) and sponge gourd (*p* 0.0052); and significant for spinach (*p* 0.0102) and coriander (*p* 0.0129).

### 2.3. Estimation of the Safety of the Soil/Plant System in the Study Crops

CLI was calculated to assess the soil contamination status. A CLI value ≥1 indicates contamination [46]. In this study, CLI values 1.93, 0.027, 0.043, 0.026, 0.043, and 0.005 were calculated for Cd, Cr, Co, Ni, Mn, and Pb, respectively. Except for Cd, CLI values for Cr, Co, Ni, Mn, and Pb are <1. The order of increasing contamination factor of metals was Cd > Mn > Cr > Co > Ni > Pb. 

**Figure 1:**
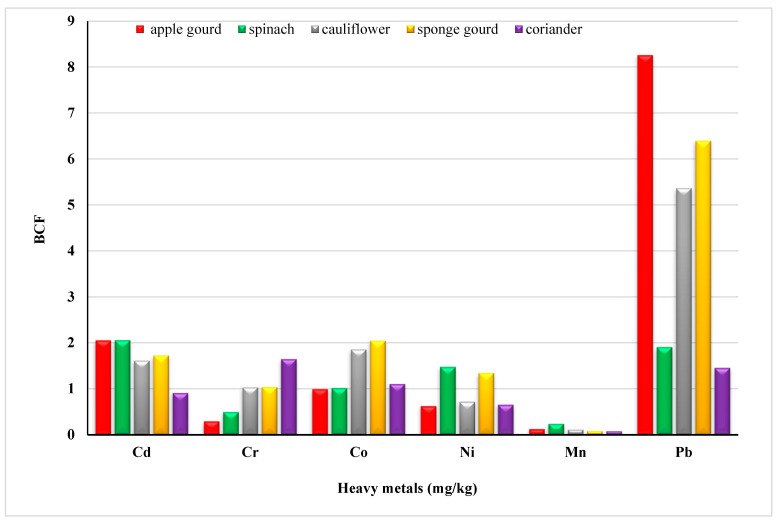
Bio-concentration factor of heavy metals in vegetables.

BCF is important to assess the HRI of soils irrigated with wastewater. Metal pollution in soil and vegetables, by themselves, provide limited information on health risks. However, BCF, from soil to crops, examines the human exposure to metals through the food chains [47,48]. The transfer of heavy metals from soils to vegetables was calculated and displayed in Figure 1. The mean transfer factor of heavy metals ranged from 0.90 to 2.05 for Cd; 0.29 to 1.64 for Cr; 0.99 to 2.04 for Co; 0.62 to 1.47 for Ni; 0.079 to 0.23 for Mn and 1.45 to 8.45 for Pb, respectively. Significant differences in BCF observed can be related to the heavy metal binding capacity to different plant tissues [49] or sorption onto other interfaces. Bose and Bhattacharyya (2008) have reported an increase in the uptake of Pb, Cr, and Cu in wheat plants [50].

**Figure 2:**
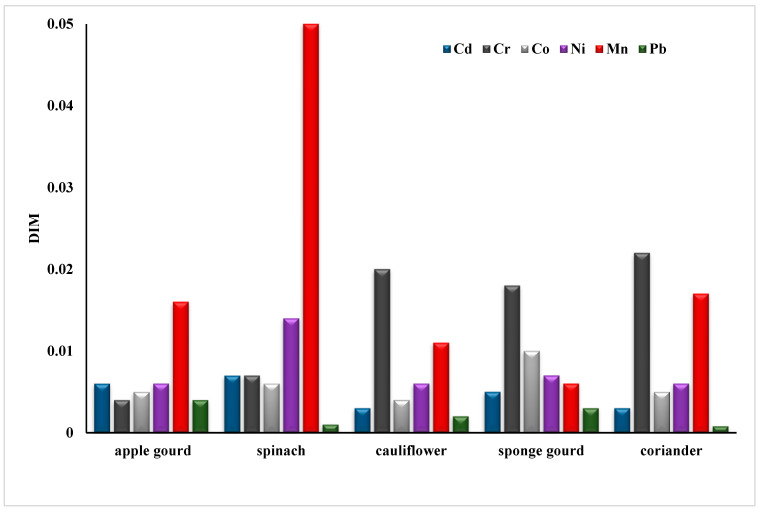
Daily intake of heavy metals in vegetables.

The transfer factor between the different metals in apple gourd was Pb (8.45) > Cd (2.05) > Co (0.99) > Ni (0.62) > Cr (0.29) > Mn (0.12). In cauliflower, it was Pb > Co > Cd > Cr > Ni > Mn. In sponge, gourd was Pb > Co > Cd > Ni > Cr > Mn. As per reported data Pb, Cd and Cr have comparable and high transfer rates. Moreover, the order of the transfer factor for Mn in spinach (Cd > Pb > Ni > Co > Cr > Mn) and coriander (Cr > Pb > Co > Cd > Ni > Mn) shows that the probability of Mn transfer is the lowest among all other metals. To quantify the potential risk to human health in the area, the daily intake of metals (Figure 2) and health risk index (Table 3) were calculated. Oral reference dose (RFD) is the maximum exposure that an individual receives throughout a lifetime without any danger; RFD values for some toxic metals in this study were 4.00 × 10^−2^, 3.00 × 10^−1^, 4.00 × 10^−3^, and 1.00 × 10^−3^ mg/Kg/day, respectively (FAO/WHO (Codex Alimentarious Commission, 2013). A greater risk to the population exists if HRI = 1, otherwise, the exposed population is at low risk [51].

All samples assessed had a higher health risk value associated with Cd, followed by Cr (details shown in Table 3). Apple gourds also showed a high value of health risk index (1.054) for Pb as compared to the safe limit. Therefore, the population is at higher risk for Cr, Cd, and Pb because their values were above 1.0. 

## 3. Materials and Methods

### 3.1. Description of Study Area

The study was conducted in Chenab-Nagar, Chiniot, Pakistan, located on the east bank of the Chenab river (31.307 N, 72.328 E). The annual mean temperature in Chiniot is 30.98 °C, 10.09% greater than Pakistan’s averages. Chiniot naturally receives approximately 22.16 mm of precipitation and has 47.41 rainy days every year.

### 3.2. Sample Collection and Preparation

#### 3.2.1. Vegetables

The vegetables selected for the study were spinach, sponge gourd, cauliflower, coriander, and apple gourd. A total of fifteen vegetables (fifteen specimens for each type) were collected in Chenab, where they had been cultivated with sewage water as usual. These samples were cut into their edible part, stem, and leaves. They were washed with distilled water, and dried in the oven for two days at 70 °C. The dried samples were homogenized, crushed, and ground to powder form using a mortar and pestle. Each vegetable sample was prepared in a 250 mL conical flask by digesting them individually (2.0 g) with 20 mL of the concentrated HNO_3_ (70% trace metal analysis quality, purchased from Merck KGaA, Darmstadt, Germany), on a hot plate for 2 to 3 h at 70 °C until complete digestion and appearance of a colorless solution in the flask. Then, the contents of the flask were cooled down at room temperature and filtered using Whatman No. 42 filter paper twice to ensure the removal of all particles. Finally, the filtrate was brought to 100 mL with the addition of double distilled water. Similarly, a blank was prepared by digesting 20 mL of the concentrated HNO_3_ in an empty flask for 2 to 3 h at 70 °C, filtering off particles and bringing the final volume of filtrate sample to 100 mL with double distilled water.

#### 3.2.2. Soil 

Composite soil samples were taken from the first 10 cm (depth) where vegetables were irrigated by sewage wastewater. Soil samples were oven-dried, sieved (2mm cut off), and processed in numbered polythene sampling bags. Non-soil particles such as gravel, organic debris, rocks, stones, and wooden pieces were removed manually from the soil samples. Soil samples were prepared in a 250 mL conical flask by digesting it (2.0 g) with 20 mL of the concentrated HNO_3_ on a hot plate for 2 to 3 h at 70 °C, filtering off and making the final volume of filtrate 100 mL with double distilled water. 

#### 3.2.3. Sewage Water

Sewage water was collected in polyethylene bottles that had been soaked and washed thoroughly with 10% HNO_3_ followed by deionized water respectively. Sewage water (100 mL) was mixed with 20 mL of 70% trace metal analysis HNO_3_ in a 250 mL conical flask, and placed on a hot plate for 2 to 3 h at 70 °C until a colorless solution was obtained after digestion. The digestate was left to reach room temperature and filtered, twice, using a Whatman No. 42 filter paper to remove dust particles. Finally, the filtrate volume was brought to 100 mL with double distilled water.

### 3.3. Analytical Procedures

Each vegetable and soil sample (2 g) was digested with 20 mL of the concentrated (70%) trace metal analysis HNO_3_ on a hot plate for 2–3 h at 70 °C. The next treatment steps follow the procedure described for sewage water. Standard calibration solutions of metals were prepared after successive dilutions from standard stock solution (10 µg/mL) of Cd, Cr, Co, Ni, Mn, and Pb, obtained from Ultra Scientific (North Kingstown, USA). A calibration solution was prepared in the range of 0.001 µg/mL to 10 µg/mL in 10% nitric acid in water. The metals in the digested and diluted samples were analyzed with atomic absorption spectroscopy (model AA-6300 Shimadzu, Kyoto, Japan). Its operating was slit width 0.6 ± 01 nm, burner height 7.0–9.0 mm, lamp current 8–10 mA, air: acetylene (15:2) at flow rate ~2 L/min. Further details are given in Table 4. Each batch of samples and blanks was analyzed in triplicate.

### 3.4. Statistical Method

A two-way Analysis of Variance (ANOVA) determined the statistical significance (α = 0.05) of metals found in the different vegetables. This assessment was carried out with Statistical Package for the Social Sciences (SPSS) software. Tuckey’s test was conducted on the mean ranks to determine the significance among all treatments (*p*, 0.05). Scheffe’s test was chosen as a post hoc test because it allows multiple comparisons. 

### 3.5. Calculation of Indices Related to the Safety of the Soil/Plant System in the Study Crops 

The Contamination Load Index (CLI) in soil, Bioconcentration Factor (BCF) in plants, Daily Intake of Metals (DIM), and Health Risk Index (HRI) were calculated as follows:

#### 3.5.1. CLI

To estimate the metal accumulation status in the study soil, CLI was calculated as indicated in (1) [32]. The reference trace metal values in soil for Cd, Cr, Co, Ni, Mn, and Pb are 1.49, 9.07, 8.39, 9.06, 44.19, and 56.90 mg/Kg, respectively.

CLI = Concentration of metal in soil/Reference concentration of metal in soil(1)

#### 3.5.2. BCF

BCF informs about the transition of metals from soil to plants. It was calculated as proposed elsewhere [27] and specified in (2).

BCF = C _veg_ ÷ C_soil_ (2)

where C_veg_ = metal accumulation (mg metal/Kg fresh mass plant tissues), and

C_soil_ = metal concentration (mg metal/Kg soil dry weight) 

#### 3.5.3. DIM

DIM estimates consumers’ health risks. It was calculated as proposed elsewhere [52] and specified in (3):

DIM = C _metal_ × C _factor_ × C _intake_ ÷ B _weight_ (3)

where, C_metal_ = concentration of metal in vegetables (mg/Kg), 

C_intake_ = daily food intake, and B_weight_ represents average body mass. 

C_factor_ = conversion factor (0.085) is used for the transformation of the fresh-weight of vegetables to dry weight. 

#### 3.5.4. HRI

HRI is an indicator of health threat for those consuming contaminated food. It is calculated as the ratio of DIM in crops with an oral reference dose [20].

HRI = DIM ÷ Oral reference dose(4)

Reference oral dose values for Cr, Ni, Pb, Cd and Mn are 1.5, 0.02, 0.004, 0.001 and 0.033 (mg/Kg bw/day), respectively (US-EPA IRIS) [53].

## 4. Conclusions

Heavy metals (Cd, Cr, Co, Ni, Pb, and Mn) have been quantified in a wastewater stream commonly used for the irrigation of vegetables in Chenab Nagar, Chiniot, Pakistan. Among the study metals, the concentration of Cr (0.24 mg/L) and Co (0.78 mg/) exceeded safe levels, and Cd (0.014 mg/L) was not significantly different (*p* 0.05) than the maximum safe limit as per FAO/WHO guidelines. If the cultivation of vegetables remains using similarly contaminated wastewater, translocation, and accumulation of heavy metals especially Cd, Cr, and Pb may occur from water to plants and livestock (animals and human beings). This may pose severe health risks, in particular with Cr from which the daily intake is high. This study has also found that coriander and cauliflower contribute to such risk, and in general Future work should gain more understanding of the influx of Cr, Co, and Cd in Chenab Nagar wastewater to reduce contamination in the water and crops receiving such water. Furthermore, Cd, Cr, and Pb should be further monitored in derived products from the study crops and livestock (e.g., milk, meat, leafy vegetables) to detect possible food safety issues. Irrigation with wastewater generated in Pakistan is suitable and it needs to be treated (at least to reduce heavy metal pollution) before its reuse.

## Figures and Tables

**Table 1:** Concentrations of heavy metals in wastewater used for irrigation and in irrigated soil. The maximum limits of metals in soil and wastewater regarded as safe have been included.

Metals	Wastewater Used for Irrigation (mg/L)	Maximum Limit of Heavy Metals (mg/L) in Wastewater Water to be Used in Irrigation [33]	Soil (mg/Kg)	Maximum (Safe) Limit of Heavy Metals in Soil (mg/Kg) [29]
Cd	0.014	0.01	0.58	3
Cr	0.240	0.10	2.43	100
Co	0.780	0.10	0.83	-
Ni	0.007	0.20	1.76	50
Mn	0.003	0.20	3.72	-
Pb	nd	5.00	0.11	2000

nd, not detected, -, not given.

**Table 2:** The quantified concentration of heavy metals in vegetables and corresponding FAO/WHO maximum permissible limits.

Vegetables	Edible Parts	Cd (Mean mg/kg ± sd)	FAO/WHO Maximum Permissible Limit (Cd, mg/kg)	Cr (Mean mg/kg ± sd)	FAO/WHO Maximum Permissible Limit (Cr, mg/kg)	Co (Mean mg/kg ± sd)	FAO/WHO Maximum Permissible Limit (Co, mg/kg)	Ni (Mean mg/kg ± sd)	FAO/WHO Maximum Permissible Limit (Ni, mg/kg)	Mn (Mean mg/kg ± sd)	FAO/WHO Maximum Permissible Limit (Mn, mg/kg)	Pb (Mean mg/kg ± sd)	FAO/WHO Maximum Permissible Limit (Pb, mg/kg)
Apple gourd	Leaves	1.20 ± 0.02	0.10	0.72 ± 0.06	-	0.83 ± 0.03	-	1.11 ± 0.01	-	4.63 ± 0.02	-	0.93 ± 0.03	0.3
	Stem	0.67 ± 0.001	-	1.20 ± 0.01	-	1.77 ± 0.01	-	1.72 ± 0.03	-	2.65 ± 0.01	-	0.14 ± 0.01	-
	Fruit	1.20 ± 0.04	0.05	1.20 ± 0.03	2.3	0.85 ± 0.04	50	2.61 ± 0.06	67.9	8.65 ± 0.05	500	0.21 ± 0.02	0.1
Spinach	Leaves	1.20 ± 0.03	0.10	1.21 ± 0.02	2.3	0.85 ± 0.01	50	2.61 ± 0.11	67.9	8.65 ± 0.06	500	0.21 ± 0.02	0.3
	Stem	1.00 ± 0.001	-	1.50 ± 0.01	-	0.74 ± 0.03	-	2.29 ± 0.01	-	6.10 ± 0.01	-	0.31 ± 0.01	-
	Fruit	0.70 ± 0.02	0.05	3.45 ± 0.03	-	0.80 ± 0.02	-	1.23 ± 0.06	-	1.81 ± 0.05	-	0.36 ± 0.09	0.1
Cauliflower	Leaves	0.94 ± 0.06	0.10	2.49 ± 0.01	-	1.55 ± 0.04	-	1.26 ± 0.06	-	4.16 ± 0.27	-	0.59 ± 0.01	0.3
	Stem	0.53 ± 0.01	-	2.26 ± 0.20	-	1.03 ± 0.10	-	0.88 ± 0.02	-	1.22 ± 0.01	-	0.14 ± 0.01	-
	Fruit	0.97 ± 0.04	0.05	2.91 ± 0.01	2.3	2.08 ± 0.10	50	1.13 ± 0.03	67.9	1.15 ± 0.18	500	0.53 ± 0.09	0.1
Sponge gourd	Leaves	1.01 ± 0.09	0.10	2.52 ± 0.01	-	1.71 ± 0.20	-	2.37 ± 0.04	-	3.18 ± 0.52	-	0.70 ± 0.02	0.3
	Stem	0.73 ± 0.05	-	2.52 ± 0.04	-	1.16 ± 0.01	-	2.57 ± 0.03	-	0.56 ± 0.01	-	0.26 ± 0.01	-
	Fruit	0.53 ± 0.04	0.05	4.00 ± 0.03	2.3	0.92 ± 0.04	50	1.16 ± 0.05	67.9	2.97 ± 0.18	500	0.16 ± 0.001	0.1
Coriander	Leaves	0.53 ± 0.04	0.10	4.00 ± 0.06	2.3	0.92 ± 0.03	50	1.16 ± 0.03	67.9	2.97 ± 0.06	500	0.16 ± 0.03	0.3
	Stem	0.38 ± 0.01	-	3.80 ± 0.05	-	1.60 ± 0.01	-	1.30 ± 0.04	-	1.70 ± 0.18	-	0.11 ± 0.03	-
	Fruit	1.04 ± 0.03	0.05	0.64 ± 0.01	-	0.76 ± 0.01	-	0.80 ± 0.03	-	3.19 ± 0.42	-	0.66 ± 0.03	0.1

sd, standard deviation (*n* = 3); -, not described.

**Table 3:** Calculated Health Risk Index found for the different study vegetables.

Vegetables	Heavy Metals					
	Mn	Cd	Pb	Co	Ni	Cr
Apple gourd	0.44	11.96	1.05	0.22	0.26	1.25
Spinach	1.21	13.85	0.33	0.24	0.75	2.30
Cauliflower	0.25	8.05	0.58	0.23	0.35	6.61
Sponge gourd	0.16	11.25	0.85	0.60	0.32	5.57
Coriander	0.42	6.10	0.26	0.26	0.33	7.67

**Table 4:** Summary of operating conditions employed for the analysis of metals with atomic absorption spectrophotometer.

Parameters	Cd	Cr	Co	Ni	Mn	Pb
Wavelength (nm)	228.8	422.7	395.4	232.0	279.5	283.3
Slit width (nm)	0.6	0.6	0.6	0.7	0.2	0.6
Lamp current (mA)	8.0	8.0	10	8.0	10	10
Air flow rate (L/min)	15	15	15	15	15	15
Acetylene flow rate (L/min)	2.0	2.6	1.8	2.0	1.8	2.2
Burner height (mm)	9.0	7.0	7.0	7.0	7.0	7.0

## Data Availability

Not applicable.

## Supplementary Materials

The following supporting information can be downloaded at: https://www.mdpi.com/article/10.3390/molecules28031371/s1, Table S1: Two factor analysis of variance for concentration (mg/kg) of heavy metals in collected vegetable samples.

## Sample Availability

Samples of the compounds are available from the authors.
