# Supplementary material for: Wastewater-Irrigated Vegetables Are a Significant Source of Heavy Metal Contaminants: Toxicity and Health Risks
Source: Molecules. 2023 Feb 1;28(3):1371. doi: 10.3390/molecules28031371 (PMC9919308; doi:10.3390/molecules28031371)
Supplement: Supplementary file 1 [file molecules-28-01371-s001.zip › molecules-2092119-supplementary.pdf]

Table S1. Two factor analysis of variance for concentration (mg/kg) of heavy metals in collected vegetable samples.

Vegetables	Source of variation	*SS*	*df*	*MS*	*F*	Sig.
Apple gourd	Corrected models	205.68	17	12.10	9565.94	0.00
	Parts	12.14	2	6.07	4798.99	0.00
	Metals	142.28	5	28.46	22498.47	0.00
	Error	0.05	36	0.001		
	Total	380.37	54			
Spinach	Corrected models	243.49	17	14.32	6778.69	0.00
	Parts	10.23	2	5.12	2421.82	0.00
	Metals	159.47	5	31.89	15094.86	0.00
	Error	0.08	36	0.002		
	Total	447.66	54			
Cauliflower	Corrected models	51.11	17	3.01	285.14	0.00
	Parts	6.09	2	3.05	289.07	0.00
	Metals	30.15	5	6.03	571.94	0.00
	Error	0.38	36	0.01		
	Total	162.61	54			
Sponge gourd	Corrected models	66.69	17	3.92	193.51	0.00
	Parts	3.41	2	1.70	84.03	0.00
	Metals	44.27	5	8.85	436.76	0.00
	Error	0.73	36	0.02		
	Total	207.87	54			
Coriander	Corrected models	79.88	17	3.92	193.51	0.00
	Parts	3.442	2	1.70	84.03	0.00
	Metals	48.81	5	8.85	436.76	0.00
	Error	0.46	36	0.02		
	Total	187.59	54			

SS, total sum of squares; *df*, number of degrees of freedom; *M*, mean square, *F*-distribution.
